# Proliferative glomerulonephritis with monoclonal IgG deposits: a unique case with a clinical course of over 46 years

**DOI:** 10.1186/s12882-023-03178-2

**Published:** 2023-04-25

**Authors:** Tyler James, Marjan Afrouzian, Luan Truong, Omar Aleter, John Badalamenti, Hania Kassem

**Affiliations:** 1 School of Medicine, University of Texas Medical Branch, Galveston, TX USA; 2 Department of Pathology, University of Texas Medical Branch, Galveston, TX USA; 3 Department of Pathology, Methodist Hospital, Houston, TX USA; 4 Farah Medical Campus, Amman, Jordan; 5 Division of Nephrology and Hypertension, Department of Internal Medicine, University of Texas Medical Branch, Galveston, TX USA

**Keywords:** Proliferative glomerulonephritis, Prolonged follow-up, Monoclonal deposits, IgG, Case report

## Abstract

**Background:**

Proliferative glomerulonephritis with monoclonal IgG deposits (PGNMID) is a rare entity first described in 2004. We present a case of PGNMID with recurrent hematuria and nephrotic range proteinuria with three biopsies over 46 years.

**Case presentation:**

A 79-year-old Caucasian female presents with a history of two separate episodes of biopsy-proven recurrent GN over a course of 46 years. Both biopsies from 1974, and 1987 were reported as membranoproliferative GN (MPGN). The patient presented in 2016 for the third time with symptoms of fluid overload, slight worsening in renal function, and proteinuria along with glomerular hematuria. A third kidney biopsy was performed, and the final diagnosis was proliferative glomerulonephritis with monoclonal IgG/κ deposits.

**Conclusion:**

With three renal biopsies obtained over 46 years, our case opens a unique window into the natural history of PGNMID. The three biopsies demonstrate the immunologic and morphologic evolution of PGNMID in the kidney.

## Background

Monoclonal gammopathy of renal significance (MGRS) is a recently-described term that encompasses several diseases. It is defined as a clonal proliferative disorder producing nephrotoxic monoclonal immunoglobulins that does not meet criteria for treatment of a specific malignancy or infection [1]. The spectrum of MGRS renal lesions is broad, and glomerulopathies are featured either by organized or non-organized deposits [2, 3].

In 2004, Nasr et al.described a unique form of glomerular injury associated with monoclonal IgG deposition, which was termed “proliferative glomerulonephritis with monoclonal IgG deposits” (PGNMID) [4]. PGNMID is featured by glomerular monoclonal IgG deposits and granular deposits by electron microscopy (EM) [4, 5]. Since then, additional cases of PGNMID have been described.

To date, most cases of PGNMID have not had prolonged follow-up, and thus the natural history of PGNMID is not well-understood. We report a patient with PGNMID with three renal biopsies over 46 years of follow-up.

## Case presentation

A 79-year-old Caucasian female presented in 2016 with symptoms of fluid overload, progressive fatigue, worsening hypertension, and edema. Past medical history was significant for hypertension, gouty arthritis, gastroesophageal reflux disease, and previous history of MPGN. Medications included labetalol, colchicine, furosemide, sertraline, and esomeprazole. Her blood pressure on presentation was 178/103 mmHg. Physical examination showed facial swelling and 1 + bilateral lower extremity edema up to the mid-shin. Laboratory results showed a serum albumin of 3.1 g/dL (reference range: 3.5—5.0 g/dL), BUN of 33 mg/dL (reference range: 7—23 mg/dL), serum creatinine of 1.3 mg/dL (reference range: 0.50—1.04 mg/dL), and 3 + protein in urine. ESR was 34 mm/HR (reference range: 0—20 mm/HR). Urine microscopy showed persistent glomerular hematuria. The urine protein/creatinine ratio was 7.5 g/g. Serum and urine protein electrophoresis demonstrated hypogammaglobulinemia, selective proteinuria, and no M-spike. Serum cryoglobulin was negative. Hepatitis panel was also negative. C4 complement was 24 mg/dL (reference range: 20—59 mg/dL) and C3 was 107 mg/dL (reference range: 86—184 mg/dL). Later laboratory studies showed a minimally elevated κ/λ free-light-chain-ratio of 1.73 (reference range: 0.26—1.65).

**Fig. 1:**
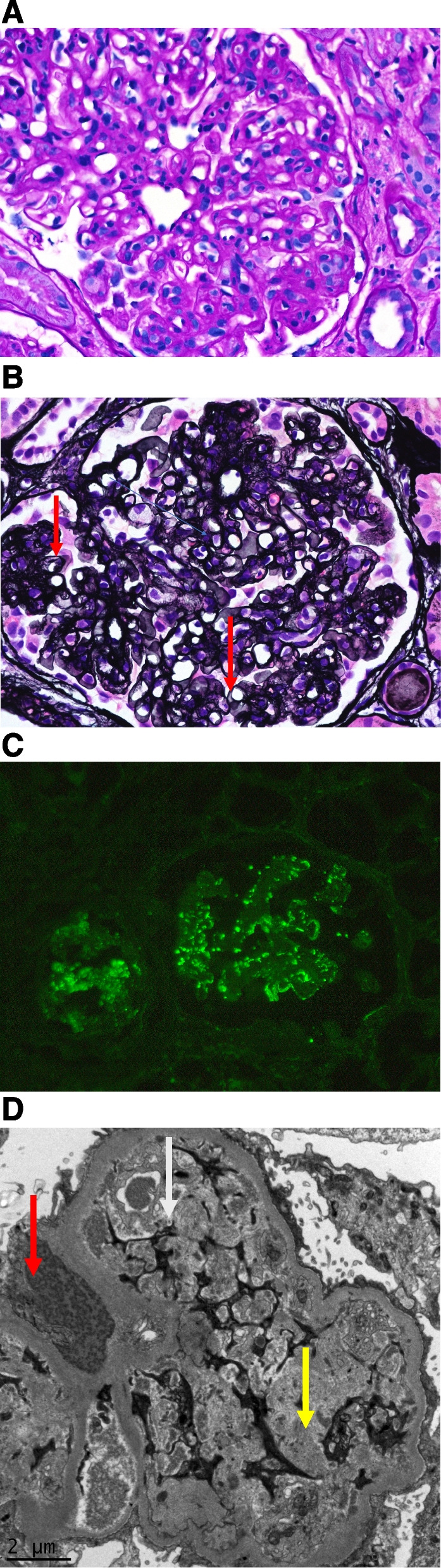
**A** Glomerulus showing endocapillary proliferation associated with increase in mesangial matrix and cellularity (PAS, × 400). **B** Glomerulus showing small capillary lumina, thickened GBMs and rare double contours (arrows) (PAMS, × 400). **C** Glomeruli showing mesangial deposits of IgG3 by IF (IgG3, × 200). **D** A segment of a glomerulus showing irregular and thickened GBM with one large sub-epithelial variegated electron dense deposits (hump) (red arrow), mesangial interposition (white arrow) and expanded mesangium containing electron dense deposits (yellow arrow) (EM, × 4000)

Detailed review of her history revealed:

- **First renal biopsy**
  She reportedly had a presentation consistent with acute glomerulonephritis and a kidney biopsy in 1974 was consistent with membranoproliferative glomerulonephritis (MPGN). She was managed conservatively and no further information was available regarding the clinical presentation or pathology findings.
- **Second renal biopsy**
  In 1987, she again presented with persistent microscopic hematuria and proteinuria, but with well-preserved renal function; a second biopsy was done.
  Light microscopy (LM) demonstrated diffuse mesangial proliferation. Glomerular capillary basement membranes (GBMs) were normal thickness. Capillary loops exhibited focal and segmental thickening and splitting. Inflammatory cells composed of polymorphonuclear leukocytes (PMNs) and monocytes were found within isolated capillary lumina. There was mild interstitial edema accompanied by mild interstitial fibrosis and tubular atrophy.
  Immunofluorescence (IF) showed granular deposits of C1q and C3 diffusely in the mesangium. Fine and coarse granular deposits of IgG, C3, and C1q were observed along GBMs and adjoining mesangial areas focally and segmentally. Tubular epithelial cell absorption droplets stained with IgG, C3, and albumin. Globular deposits of IgM and granular deposits of C3 were observed in arterial and arteriolar walls. No IgG subtyping was performed at the time.
  By EM, GBMs were normal thickness with minimal wrinkling and irregularity with an increase in the mesangial matrix. Mesangial interposition and new basement membrane formation on the endothelial aspect was observed. Large subendothelial and mesangial electron-dense-deposits (EDD) were present. There were no subepithelial or intramembranous deposits. Foot processes were focally effaced.
  The diagnosis was focal, segmental MPGN and she was managed conservatively, with the main focus being blood pressure control, gout control, and lipid management. The patient was on and off on calcium channel blockers, beta blockers, and a thiazide diuretic but was not at the time placed on a RAAS blocker likely because she had a spontaneous remission.
- **Third renal biopsy**
  She agreed to a third kidney biopsy after her previously described presentation in 2016 and due to decreased kidney function.
  LM demonstrated 9 glomeruli; one glomerulus was globally sclerotic, and remaining glomeruli were enlarged. Six glomeruli showed thickened GBMs, mild endocapillary proliferation (Fig. 1A) and double contours on silver stain (Fig. 1B). Three glomeruli showed segmental sclerosis with capsular adhesion. Other remarkable findings included hyaline protein droplets within tubules, mild tubular atrophy, and diffuse interstitial edema with moderate interstitial infiltration of PMNs. Arterioles showed mild hyalinosis.
  IF studies showed severe granular subepithelial staining only for IgG, C3, and κ. IgG subtyping revealed strong positivity for IgG3 (Fig. 1C).
  By EM, the irregular GBM contained EDD and resorbed deposits. Spikes and intramembranous deposits were identified. Huge subepithelial humps containing variegated deposits were present (Fig. 1D). Mesangial interposition was observed in some capillaries. The mesangium was expanded, containing unorganized EDD. No organized deposits were seen, and foot processes were effaced in many areas.
  The diagnosis of PGNMID with IgG3 and monotypic κ-light-chains was made.

### Treatment and follow-up

The third renal biopsy was complicated by hematuria, a large perinephric hematoma, an acute drop in hemoglobin of 2 g/dL, and hypotension. The patient required hospitalization at the intensive care unit and had to undergo continuous bladder irrigation and an arteriogram of the right renal artery. She also had severe AKI with creatinine peaking at 3 mg/dL. The patient eventually stabilized and was discharged home. Losartan and furosemide were resumed to control edema. Due to lack of an established therapy and the patient’s reluctance, no immunosuppression was prescribed. No bone marrow biopsy was performed as neither the patient’s serum and urine electrophoresis nor the serum light chains quantification were consistent with a plasma cell dyscrasia. Kidney function continued to decline, and in May of 2017, she agreed to attempt daily prednisone therapy at 40 mg daily. However, renal function continued to decline, requiring hemodialysis in June for uremia. In 2019, she had difficulty tolerating fluid removal. Multiple interventions were attempted, however, her condition continued to worsen. She elected to stop all dialysis therapy and eventually expired in November of 2019, shortly after stopping dialysis.

## Discussion and conclusion

We report a case of PGNMID with 46 years of follow-up. The natural history and renal prognosis of PGNMID are not well-known. Nonetheless, the pathology of PGNMID has been elegantly described by Nasr et al. [5]. The predominant histologic pattern for PGNMID is that of MPGN or endocapillary proliferative glomerulonephritis [5]. There is usually granular pattern of immunofluorescence, with deposits localized to glomerular capillary walls and mesangium [5]. IgG3 deposits are the most frequently deposited immunoglobulin and are usually κ-light-chain restricted [5]. By EM, granular EDD are confined only to the glomerular compartment, and are primarily localized in the subendothelial and mesangial areas, often with foot process effacement [5].

The pathogenesis of PGNMID remains largely unknown, though nephrotoxic characteristics of IgG3 may play a mechanistic role [5]. IgG3 has been described as “nephritogenic” due to its intrinsic properties, including positive charge, high molecular weight, self-aggregability, and high complement-fixing capacity [5–7]. In addition, few patients demonstrate detectable serum M-spikes or plasma cell dyscrasias, suggesting that PGNMID arises in the course of normal immune responses with proliferation of a B-cell clone producing monoclonal IgG [5].

The prognosis of PGNMID is reportedly poor, with many patients ultimately progressing to end-stage-renal-disease (ESRD) [5]. Our patient had a relatively benign course over four decades, even though she was not treated with immunosuppression until very late in the course of disease. No light chain restriction was reported on the first two biopsies which may be due to the difference in technique. However, it is more likely that the patient had the same disease process rather than having idiopathic MPGN initially followed by PGNMID. She progressed to ESRD requiring hemodialysis 10 months after the third biopsy, which was likely due to a combination of several factors, including AKI associated with complications from her third renal biopsy (presumed to be due to hemodynamic instability and contrast exposure) in addition to the patient’s advanced age at presentation and the lack of an established treatment course for PGNMID [8, 9].

The slow progression of disease in this patient who did not receive immunosuppressive therapy suggests that PGNMID may have a better prognosis than previously described. However, cases reported in the literature may have not presented until later in the course of illness when the disease was more advanced, as PGNMID has only recently been described.

There are multiple limitations to this case report including lack of pathology data available regarding the first biopsy and lack of detailed clinical data after the first two biopsies. Furthermore, no other investigation was done to identify a potentially pathologic clone except for serum and urine electrophoresis and serum free light chains, which are negative in the majority of patients with PGNMID. However, there is low likelihood of detecting such a clone when no circulating paraprotein is found [10, 11]. As for treatment, even though no clone was detected, it has been previously shown that clone-directed therapy with agents such as bortezomib or rituximab may have a good response rate [11]. In this case, the patient was aware of lack of strong evidence to support any specific approach and as a result elected to be managed conservatively.

In conclusion, our case opens a window into the natural history of PGNMID, contradicting previous literature associating PGNMID with a poor prognosis. However, early diagnosis in this case did not affect the patient’s overall outcome and is unlikely to change the course of the disease, especially since there is no established treatment course.

## Data Availability

All data generated or analyzed during this study is included in this published article.

## Abbreviations

MGRS: Monoclonal gammopathy of renal significance
PGNMID: Proliferative glomerulonephritis with monoclonal IgG deposits
EM: Electron microscopy
MPGN: Membranoproliferative glomerulonephritis
LM: Light microscopy
GBMs: Glomerular capillary basement membranes
PMNs: Polymorphonuclear leukocytes
IF: Immunofluorescence
EDD: Electron-dense-deposits
ESRD: End-stage-renal-disease
